# Predictability of arousal in mouse slow wave sleep by accelerometer data

**DOI:** 10.1371/journal.pone.0176761

**Published:** 2017-05-18

**Authors:** Gustavo Zampier dos Santos Lima, Sergio Roberto Lopes, Thiago Lima Prado, Bruno Lobao-Soares, George C. do Nascimento, John Fontenele-Araujo, Gilberto Corso

**Affiliations:** 1 Universidade Federal do Rio Grande do Norte, Escola de Ciências e Tecnologia, Natal, RN, Brazil; 2 Universidade Federal do Rio Grande do Norte, Departamento de Biofísica e Farmacologia, Natal, RN, 59078-970, Brazil; 3 Universidade Federal do Paraná, Departamento de Física, Curitiba, PR, 81531-980, Brazil; 4 Universidade Federal do Rio Grande do Norte, Departamento de Engenharia Biomédica, Natal, RN, 59078-970, Brazil; 5 Associate Laboratory for Computing and Applied Mathematics, Brazilian National Institute for Space Research, São José dos Campos, SP 12227-010, Brazil; 6 Universidade Federal dos Vales do Jequitinhonha e Mucuri, Instituto de Engenharia, Ciência e Tecnologia, Janaúba, MG, 39440-000, Brazil; 7 Universidade Federal do Rio Grande do Norte, Departamento de Fisiologia – 59056-450, Natal, RN, Brazil; Imperial College London, UNITED KINGDOM

## Abstract

Arousals can be roughly characterized by punctual intrusions of wakefulness into sleep. In a standard perspective, using human electroencephalography (EEG) data, arousals are associated to slow-wave rhythms and K-complex brain activity. The physiological mechanisms that give rise to arousals during sleep are not yet fully understood. Moreover, subtle body movement patterns, which may characterize arousals both in human and in animals, are usually not detectable by eye perception and are not in general present in sleep studies. In this paper, we focus attention on accelerometer records (AR) to characterize and predict arousal during slow wave sleep (SWS) stage of mice. Furthermore, we recorded the local field potentials (LFP) from the CA1 region in the hippocampus and paired with accelerometer data. The hippocampus signal was also used here to identify the SWS stage. We analyzed the AR dynamics of consecutive arousals using recurrence technique and the determinism (DET) quantifier. Recurrence is a fundamental property of dynamical systems, which can be exploited to characterize time series properties. The DET index evaluates how similar are the evolution of close trajectories: in this sense, it computes how accurate are predictions based on past trajectories. For all analyzed mice in this work, we observed, for the first time, the occurrence of a universal dynamic pattern a few seconds that precedes the arousals during SWS sleep stage based only on the AR signal. The predictability success of an arousal using DET from AR is nearly 90%, while similar analysis using LFP of hippocampus brain region reveal 88% of success. Noteworthy, our findings suggest an unique dynamical behavior pattern preceding an arousal of AR data during sleep. Thus, the employment of this technique applied to AR data may provide useful information about the dynamics of neuronal activities that control sleep-waking switch during SWS sleep period. We argue that the predictability of arousals observed through DET(AR) can be functionally explained by a respiratory-driven modification of neural states. Finally, we believe that the method associating AR data with other physiologic events such as neural rhythms can become an accurate, convenient and non-invasive way of studying the physiology and physiopathology of movement and respiratory processes during sleep.

## Introduction

The sleep-wake cycle in mammals is controlled by the interactions of different neuronal systems located in distinct brain regions, including hypothalamus and brainstem [1]. This cycle is described as a process of “sleep-wake switch” that is responsible for the expression of a wakefulness phase and a complex sleep phase [2]. In different species oscillator models have been proposed to explain the dynamics of sleep-wake cycle on different time scales: from minutes to hours or days. Furthermore, the sleep phase is influenced by the homeostatic component and by circadian and ultradian rhythms [1, 3, 4].

The sleep homeostatic component can be represented by a factor which increases during the waking phase and is responsible for the induction and maintenance of sleep [4]. The corresponding physiological substrate of the homeostatic component is the delta wave sleep in rodents and slow wave sleep (SWS) in humans. In humans, this component has its major expression during the first part of sleep phase and is dramatically reduced throughout the rest of sleep duration [5]. This mechanism is responsible for the amplitude and continuity of SWS episodes. We also know that the duration of episodes and the SWS dynamics are dependent on body size and the metabolism of the species. Small animals, such as rodents, disclose polyphasic sleep profile with multiple and alternate occurrences of sleep and waking stages. More specifically, during SWS stage, several episodes of arousals occur and the subject usually returns, after a few seconds, to the previous SWS electrophysiological pattern [6–8].

In a broad sense, arousals indicate a temporary intrusion of wakefulness into sleep [9, 10], that can be characterized as elements weaved within the texture of sleep taking part in the regulation of the sleep phase [6, 11]. Arousals are associated with several physiologic records such as a rapid modification of EEG frequency, which can include theta and alpha activity, an increase in the electromyographic activity amplitude and in the cardiac frequency [12, 13]. In this work we use data from accelerometer recordings (AR), being the accelerometer placed in the mouse head to identify arousal events which are characterized by strong AR signal fluctuation. Moreover, we describe that it is possible to predict arousals by analyzing only the accelerometer signal.

Studies have been done in order to understand the temporal dynamics of arousal episodes during SWS [12, 14, 15]. In these works they investigated changes in electrophysiological signals, specially in the cortex and in the hippocampus [12, 13]. Here, we studied the temporal dynamics of arousal episodes using a body movement signal through an accelerometer device associated with the determinism quantifier (recurrence plot) [16], that results as a predictor of arousals. Furthermore, we paired the accelerometer data record with the local field potentials (LFP) signals from the CA1 hippocampus brain area to ensure that the animal is in the SWS dynamic stage.

Bioelectrical brain records have long been used to characterize sleep-stages and to identify arousal events. On the other side, accelerometer records are a recent methodology to record animal activities. By placing an accelerometer device in the head of an mouse it is possible to detect even small body activity caused by breathing, heart beating, tremors or limb movements [17]. In this work we analyzed two simultaneous physiologic records of mice: hippocampal local field potentials (LFP) from CA1 region, obtained through surgically implanted electrodes, as well as an accelerometer record, is a relatively new and non invasive tool for performing motor behavior analysis. Furthermore, we focus our attention on the dynamical characteristics of records between arousal episodes. Remarkably, for the first time we notice that arousals, during SWS stage, are preceded by an unique acceleration dynamical pattern—occurring few seconds before—in the animal body movement.

## Materials and methods

### Experimental setup

**Fig 1:**
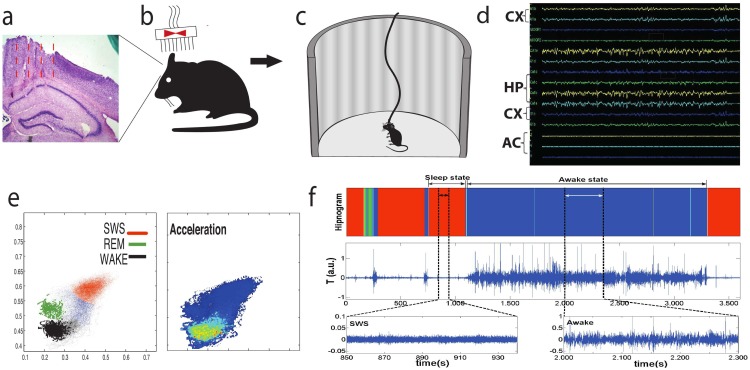
Schematic presentation of experimental procedure, data acquisition and morphological patterns in physiological dynamics associated with sleep stages and arousals. (a) Histological analysis. After all behavioral procedures and data collection, all implanted C57-BL/6J mice were perfused and brain slices were obtained in order to confirm if electrodes were targeted in M1 and S1 cortical regions and in hippocampus CA1 sub-field as an inclusion criterion. (b) Surgical Matrix Electrode Implant. Under a isofurane anesthesia a rectangle was opened in cranial bones for allowing a 16-tungsten electrode matrix to be implanted in the skull with dental cement: eight electrodes were placed in the S1/M1cortex (layers 3-5) and another eight in the CA1 sub-field of hippocampus. (c) Electrophysiology record. One week after surgery, animals were submitted to a session of 12 hours continuous recording in a round open field maze. Simultaneous LFP and Accelerometer recordings were performed in the headstage using the Plexon system. The animals were allowed to perform their natural behaviors—including the sleep-wake cycle—during the recordings. (d) LFP and Accelerometer fluctuations. All channels of hippocampus, cortex and accelerometer were displayed in real time analysis in order to get track the quality of the signal recordings. (e) State map generation. Two-dimensional behavioral state maps were generated by plotting the according to specific spectral-band ratios (see Method section) to display the stage regions: SWS(red), REM (green) and wake (black). The blue is the transition stage (left). Also accelerometer data in the state map was used to isolate wake cluster (right). Raw LFP and EMG activity were also analyzed during periods of WK, SWS, and REM sleep predicted by the two-dimensional state map. (f) A typical AR fluctuation (intermediate) associated with the hypnogram (top) generated by identification of Waking (blue), SWS (red) and REM (green) sleep cluster separation and two zooms (bottom) of the raw accelerometer signal during SWS and awake states, which were selected here for visualization.

The experiment was conducted with 5 adult male mice (2-5 months). Animals were surgically implanted with 16 chronic electrodes in the hippocampus, motor and somatosensory cortex for LFP recordings for characterizing the wake-sleep cycle as shown in Fig 1(a). After surgery, a veterinarian monitored animals twice a day. Data collection was performed during an interval of 7-14 days after surgery in order to allow animals to recover from this procedure. Since then, the animals stayed in a special home cage with a metal cover of 5 cm high in all extension to prevent helmet rupture and the food pellets were placed on the cage floor. In order to alleviate suffering, for three days after surgery animals received ad libitum paracetamol diluted in drinking water.

During data collection, three-axis accelerometer sensor (ADXL330, Analog Devices) was also attached to the headstage connector at the mouse head [Fig 1(b)], being in contact with the mouse only during the experimental measurement. The headstage was homemade and designed to have 16 channels with high input impedance amps, and compatible with system Plexon trademark (output). This experimental setup allowed simultaneous recordings of brain electrical activity (LFP) and motor activity (accelerometer). To permit free movement of the animal, the headstage and its cable were kept suspended with of a rubber band. Both electrophysiological and accelerometer signals were conditioned in the headstage and then routed to the plexon electrophysiological system. From the original 16 channels we used 14 channels being two left as spare. Six channels were implanted in the sensorimotor cortex (S1 and M1); five in the hippocampus (CA1) and three received analogical signal of the accelerometer. The accelerometer signals were low-pass filtered to have a -3dB frequency limit around 40Hz. All data acquisition was performed using a sample rate of 1 KHz.

After the surgery recovering period, the connection of the head-stage with the recording device (Plexon- INC) equipment was performed for data collection. All animals were housed in home cages in 12-12 hours light/dark schedule [Fig 1(c)]. The animals were euthanized by a lethal injection of sodium pentobarbital at the end of data collection and submitted to perfusion and histological procedures. All animals were provided by the IINN—ELS Central Biotherium. The protocol (number 08/2010) was approved by the institutional ethics committee (Comitê de Ética em Experimentação Animal do Instituto Internacional de Neurociências de Natal—Edmond and Lily Safra: IINN—ELS). Housing, surgical and behavioral procedures were in accordance with the National Institutes of Health guidelines for animal experimentation.

#### Experimental setup: Identification of deep SWS stage and arousal episodes

Spectral analysis of sleep-awake cycle was used to identify and quantify occurrence of wake, paradoxical sleep or rapid eyes movement (REM) and slow wave-sleep (SWS) states, using Plexon system for multiple LFP channel processing [see Fig 1(d) and 1(e)]. On-line LFP hypnogram maps for the characterization of awake and REM/SWS sleep states were employed [18]. In order to perform the state-map, we selected hippocampal and cortical channels that present electrophysiological activities and not show visible artifact (in the average 3 of 5 channels were selected). Furthermore, to perform the analysis comparing accelerometer data and LFP signals we used specific signals from accelerometer and from hippocampus, respectively. Slow-wave sleep states were behaviorally defined as presenting stillness with eyes closed and large-amplitude slow hippocampal oscillations [see Fig 1(d)].

Animal behavior and LFP were continuously observed in real time for 12 hours. For performing the analysis we discarded the first recording hour (related to animal environmental habituation) and used an uninterrupted 11 hours period. Recordings started at 12 p.m. (lights on) and were extended until midnight (lights off at 19:00h). Animals displayed during the experimental procedure the following normal behavioral states: wake (WK, with a general characteristic of active exploration of the environment, whisking and hippocampal alpha/theta rhythm), Slow Wave Sleep (SWS, characterized by stillness, closed eyes and large-amplitude slow hippocampal rhythm) and finally paradoxical sleep/REM (REM stage, in general, with stillness, prolonged whisking, eyes closed and hippocampal theta rhythm).

The identification of arousals was performed using the AR. An arousal is a fluctuation of the AR that has the minimal duration of 0.3s and an amplitude of at least three times the standard-deviation of signal background. In some cases arousals come not as a single peak but in a train of subsequent peaks (less than 0.5 sec of interval) that we identify as a single arousal. Previous works evidenced the occurrences of arousals in SWS in rodents with similar features to which we defined here [19–21]. However, in this work, we study arousal events based on the accelerometer fluctuation, rather than on the electromyography or LFP criteria which were used in those studies.

Arousals were selected from the SWS cluster of state maps. The SWS records presented large-amplitude slow hippocampal rhythm and low amplitude in the accelerometer record. Spectral analysis of sleep-wake cycle was used to identify and quantify occurrence of wake, paradoxical sleep or rapid eyes movement (REM) and slow wave-sleep (SWS) states, using Plexon system formal tiple LFP channel processing [see Fig 1(c)]. On-line LFP spectral maps for the characterization of waking and REM/SWS sleep states were employed [11, 17, 18, 22].

### Data treatment

The accelerometer output consists in three components of the acceleration vector *a*(*i*) = (*a*_*x*_, *a*_*y*_, *a*_*z*_). To perform the mathematical analysis we combine the vector components in a single quantity similar to the vector module at the same time that we extract the average of each vector component—here we exclude the earth gravitational acceleration to get only the inertial information from the animal movement. The new *t*(*i*) vector formed from the components of *a*(*i*) is defined as; $T=(a_{x}-{\hat{a}}_{x})+(a_{y}-{\hat{a}}_{y})+(a_{z}-{\hat{a}}_{z})$ where $\hat{a}$ is the mean of *a* [see Fig 1(e)].

### Dynamical analysis

Data analysis was performed using properties of recurrence of the trajectories. Recurrence is a fundamental property of dynamical systems, which can be exploited to characterize time series properties. The main point of recurrence analysis is the fact that its analysis can be performed using two quite basic properties of a dynamical system: (i) similar trajectories evolve in a similar way and, (ii) if a small region in the space of trajectories is visited once, probably it will be visited again and again in the future. Moreover, the frequency and the distribution in time of the visits bring valuable information about the system dynamics [16]. A powerful tool for the visualization and analysis of recurrence plots is the so called recurrence quantification analysis introduced in the late 1980’s [23]. Recurrence plots (RPs) [16, 24] are two-dimensional graphical representations of a matrix **R**_*i*,*j*_ = **Θ**(*ϵ* − ||**T**_*i*_ − **T**_*j*_||), *i*, *j* = 1, 2, …, *N*, where $T_{i}(T_{j})\in {\mathbb{R}}^{d}$ represents the reconstructed dynamical state at time *i* (*j*), *ϵ* is a predefined threshold, **Θ** is the step function (normalized), ||⋯|| stands for the Euclidean norm, and *N* is the total number of points. The RP is obtained by assigning a black (white) dot to the points for which **R**_*i*,*j*_ = 1 (0).

An important tool to explore RPs is the recurrence quantification analysis (RQA). The RQA consists of a series of measures obtained from a RP which can elucidate various aspects of the system behavior. One of the most impressive feature of these analysis is the possibility to predict stationarity in a time series [16, 24].

In the framework of RQA we have a number of quantitative diagnostics of the distribution of points in a RP. Here we cite two of them: the Recurrence Rate *RR* defined as the probability of finding a black recurrence point (for which **R**_*i*,*j*_ = 1), meaning that $RR=1/N^{2}\sum_{i,j=1;i\neq j}^{N}{\text{R}}_{i,j}$ where *N*^2^ is the total number of pixels (black or white) in a RP. We remark that the main diagonal points are excluded from the double sum, since each point is recurrent with itself.

Some quantifiers of the RPs employ the measure of diagonal lines. These are structures in a RP parallel to the main diagonal **R**_*i*,*i*_ = 1, *i* = *j* = 1, 2, …, *N*, and defined as **R**_*i*+*k*,*j*+*k*_ = 1(*j* = 1, 2, ⋯ *N*; *k* = 1, 2, ⋯, ℓ), **R**_*i*,*j*_ = **R**_*i*+ℓ+1,*j*+ℓ+1_ = 0, where ℓ is the length of the diagonal line, which occurs when a segment of a given trajectory (in phase space) runs parallel to another segment. In other words, when a RP presents a diagonal line, two pieces of a trajectory undergo for a certain time (the length of the diagonal) a similar evolution as it visit the same region of phase space at different times. This is the key idea of recurrence and thus a clear-cut signature of determinism. Accordingly, we compute *P*(ℓ) = {ℓ_*i*_; *i* = 1, 2, … *N*_ℓ_}, which is the frequency distribution of the lengths ℓ of diagonal lines, and *N*_ℓ_ is the absolute number of diagonal lines, with the exception of the main diagonal line which always exists by construction. The second quantification (RPs) used here is the Determinism that is defined as $DET=\Sigma_{l=l_{\text{min}}}^{l_{\text{max}}}lP(l)/\Sigma_{i,j=1,i\neq j}^{N}R_{i,j}$ where ℓ_min_ = 2 is the minimum length allowed for a diagonal line, whereas the maximum diagonal length is ℓ_max_ = max(ℓ, *i* = 1, 2, … *N*_ℓ_). Thus, the determinism measures the percentage of points in a RP belonging to diagonal lines.

#### Statistical analysis, definition of events and ROC curves

We define the LDA Large Determinism Amplitude for characterize the AR signal and to predict arousals. Quantitatively, a LDA event occurs when a normalized DET varies from a value higher than 0.7 (arbitrary units, *y*-axis) to a value lower than 0.2 in less than 3s. If the LDA is associated with the beginning of the arousal we define it as a success match. Complementary, if LDA is not associated with arousal onset we considered this occurrence as a failure event.

**Fig 2:**
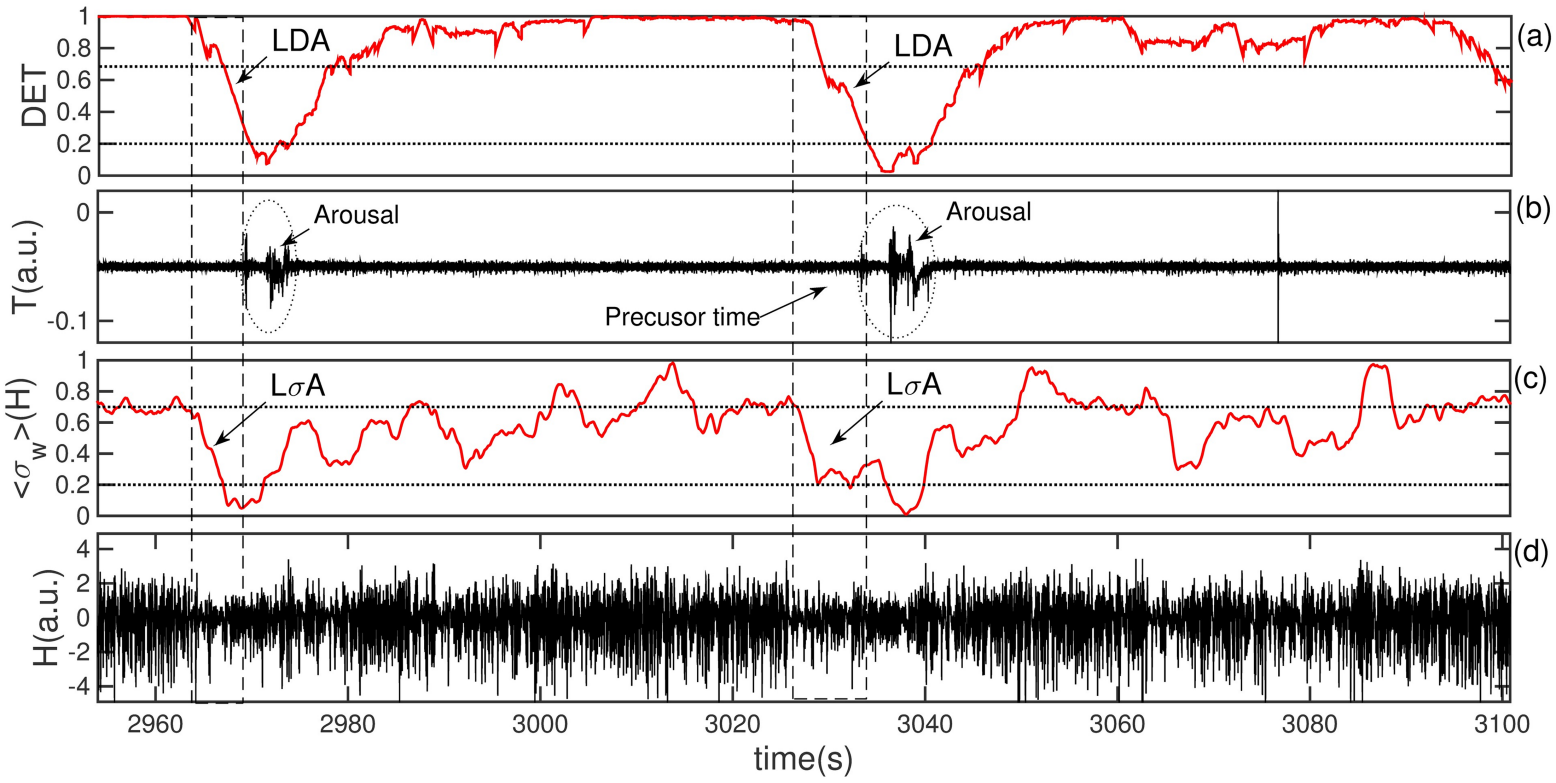
Temporal dynamics of sleep-wake cycle involving the determinism (DET) of AR—noninvasive method—and the variance (*σ*) of LFP of CA1 area—invasive method—during SWS stage. (a) The normalized temporal behavior of the determinism (DET) estimated from the AR. The same panel shows an increase and sharp decrease of DET associated with the sleep-wake cycle dynamics and arousal-burst events (dotted rectangle). The two consecutive dashed vertical lines mark the sharp decrease of DET and the following arousal event (precursor time). (b) The accelerometer record AR, the burst events represent the dynamic of arousals during the SWS sleep stage. (c) The normalized and filtered windowed variance of the amplitude of LFP CA1 activity. The panel shows the Large Variance Amplitude: *LσA* = *σ*_*max*_ − *σ*_*min*_ (defined as events that crosses the horizontal top and bottom dashed lines). We notice that the precursor time LDA of AR and *LσA* of CA1 are synchronized. (d) The raw LFP recorded at the hyppocampal CA1 area.

**Fig 3:**
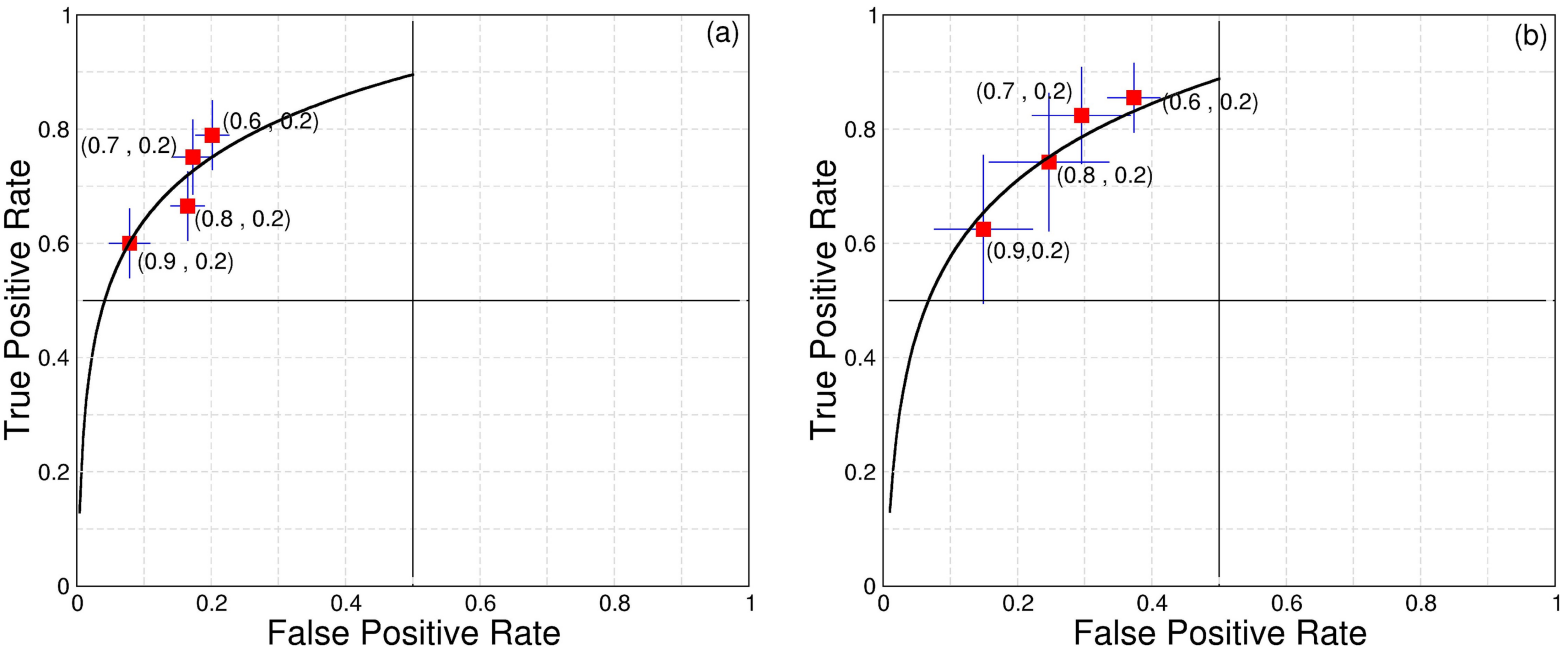
Arousal predictors ROC curve analysis during SWS stage. ROC curves for LDA (a) and L*σ*A (b), we employ the low cutoff 0.2 and explore four values of upper cutoffs. The values (0.8, 0.2) and (0.7, 0.2) for upper and lower cutoffs optimize the computation of LDA and L*σ*A as arousals predictors, these values show high true positive cases and minimize false positive cases. The solid black line is a logarithmic fitting curve used as a guide eye.

A comprehensive visualization of LDA (between two successive vertical dashed lines) is shown in Fig 2(a). Here we plot the results based on a selected threshold for upper and lower values of DET (0.7 and 0.2 respectively). These optimized thresholds were obtained performing ROC curve analysis for four sets of values. A good choice of cutoffs improves the arousal predictability of LDA and L*σ*A. To analyze how the results change as a function of upper cutoffs, Fig 3 displays a ROC curve for LDA (a) and L*σ*A (b). For all sets of upper and lower threshold a numerical algorithm evaluates all points in a time series and count all true positive and also true and false negative events. We mention that true negative was not possible in our study, since we selected a priori all events of arousals that we used in this work. Data from Fig 3 evidence that the used threshold of 0.7 for the upper cutoff optimizes our results pointing an optimal rate of true positives (sensitivity) and also a relatively low amount of false negatives (specificity). In order to guide our eyes we plot a logarithmic fitting curve.

We explore the connection between LDA and arousal in all probabilistic occurrences using both back and forth exploration in the time series. The straightforward time analysis covered: (i) LDA associated to arousal (success) and (ii) LDA not associated to arousal (failure). The backward time analysis implied in: (iii) arousal associated to LDA (success); (iv) arousal not associated to LDA (failure). This approach was used here to encompass all statistic events related to success and failure occurrences.

The performed analysis of hippocampal electric activity is simpler than the accelerometer analysis. We start with the Local Field Potential (LFP) recorded in the CA1 region of the hippocampus, than we compute the standard deviation of the LFP signal *σ* over a time window and perform a smoothing of the results (we take a subsequent average of *σ* over a time window). In order to visualize the relation between fluctuation of CA1 activity and arousal we define the mobile (windowed) standard deviation amplitude *σ*_*w*_. This quantity is defined in a similar way as the previous one: *LσA* = *σ*_*max*_ − *σ*_*min*_. In Fig 2(c) we plot a normalized *σ* to help the computation of statistics. We remark that, despite the similarity in the definition of LDA and L*σ*A the dynamics is quite diverse; in the case of LDA of DET the signal was in an high plateau and drops while in the L*σ*A case the data oscillates with high amplitude and turns to oscillate with low amplitude. To estimate success and failure events: L*σ*A preceding or not arousal, as well as L*σ*A being preceding or not by arousal we use *σ*_*max*_ ≥ 0.7 and *σ*_*min*_ ≤ 0.2. The time moving window used in LDA had a duration of 8 seconds (8192 points) and an overlapping of 8092 points; the time window in *σA* was 1 second (1000 points) with an overlapping of 900 points. All points in the two methods were positioned to the left (as past) in a way that did not compromise the prediction of points in the future.

The analyses of events of arousals, variation in determinism and decreasing in CA1 local field potential were performed over time windows of 200*s* long. The time windows are selected based on the clear indication, through hypnogram associated with accelerometer, that the mouse entered SWS state. For each window we check for arousal, LDA and L*σ*A events. We consider as an episode any event which contains at least one of these three elements.

## Results

Several processes in nature have recurrent behavior such as periodicity (e.g. circadian rhythms) [25, 26], but also irregular cyclicities (e.g.meteorologic records) [27, 28]. A recurrent state means that some dynamical states become arbitrarily close across the time, that is a fundamental property of deterministic dynamical systems [16]. Some quantifiers have been proposed to understand the recurrence phenomena and one of the most important is the *Determinism*. The recurrence analysis brings a new insight to study the body activity observed in the AR fluctuation. We show a typical analysis of the AR temporal behavior pattern in Fig 2(b), and the DET quantifier in the Fig 2(a). The raw LFP activity from CA1 brain area and its windowed variance signal—another quantifier used here—are also disclosed in Fig 2(d) and 2(c) respectively. We focus our attention here in the AR signal and the respective determinism quantifier, we also plotted the local field potential from CA1 area to indicate the transitional states in sleep-wake cycle.

A sample of raw data from all mice (the uninterrupted third hour of accelerometer, hippocampal, cortical data) is also available as raw data at supplementary material with the following archive names: S1, S2, S3, S4 and S5 Datasets.

The up and down trends of the estimated DET are highly correlated with arousal events (bursts) visualized by changes in the temporal dynamic of the AR signal, see Fig 2(b). Furthermore, as evidenced by Fig 2(a), DET signal starts to drop just before the emergence of an arousal event (LDA). Simultaneously to the change in DET behavior we observe a decreasing in the amplitude of the LFP. We estimate the change in the amplitude by computing the variance (*σ*) of the LFP. We called here the decreasing in the amplitude by a Large *σ* Amplitude (L*σ*A). Specifically, this variance is obtained by normalizing and filtering the LFP variance using the windowed standard deviation (*σ*_*w*_(*H*)) shown in Fig 2(c). Remarkably, these two mathematical normalized quantifiers decrease at the same time until they reach a minimum point which coincides with the arousal onset. Fig 2 shows the results of LDA and L*σ*A for a given upper (0.7) and lower (0.2) cutoffs. A good choice of cutoffs improves the arousal predictability of LDA and L*σ*A. To analyze how the results change as a function of upper cutoffs, Fig 3 displays ROC curves for LDA (a) and L*σ*A (b). The data show that the interval 0.7 ↦ 0.8 for the upper cutoff optimizes our results bringing good rate of true positives (sensitivity) and also a relative low values of false negatives (specificity). In order to guide the eyes we plotted logarithmic fitting curves using real data (in red) generated by ROC analysis.

The LDA captures the information of an abrupt transition in the behavior of the determinism: a rapid change in the periodicity of the AR. We use the LDA quantifier to define the precursor, an index that captures the time delay between the change in DET and the arousal event. The arousal precursor time (the time between two vertical dashed lines in the Fig 2) is employed to estimate an average duration preceding the arousal events. We evidence here that this association is observed by using AR data, discarding the requirement of invasive LFP electrode implants. We point out that the observed decrease in LFP amplitude of the CA1 area, before arousals, was already observed in others studies [29, 30] and it was successfully replicated in this paper.

Regarding the analysis for detection of LDA and arousal associations, in all situations LDA comes before the arousal in time. More specifically, AR is used to perform the following statistics: initially we identify all large DET amplitude followed by a sharp decrease (LDA) in the DET series and compute how many of them are followed by an arousal (success event). After that, we compute another success possibility: by retrograding the signal in time we count how many arousals are preceded by LDA (success event). We measured also the failure events: LDA not preceded by arousal or arousal not succeeded by LDA.

**Fig 4:**
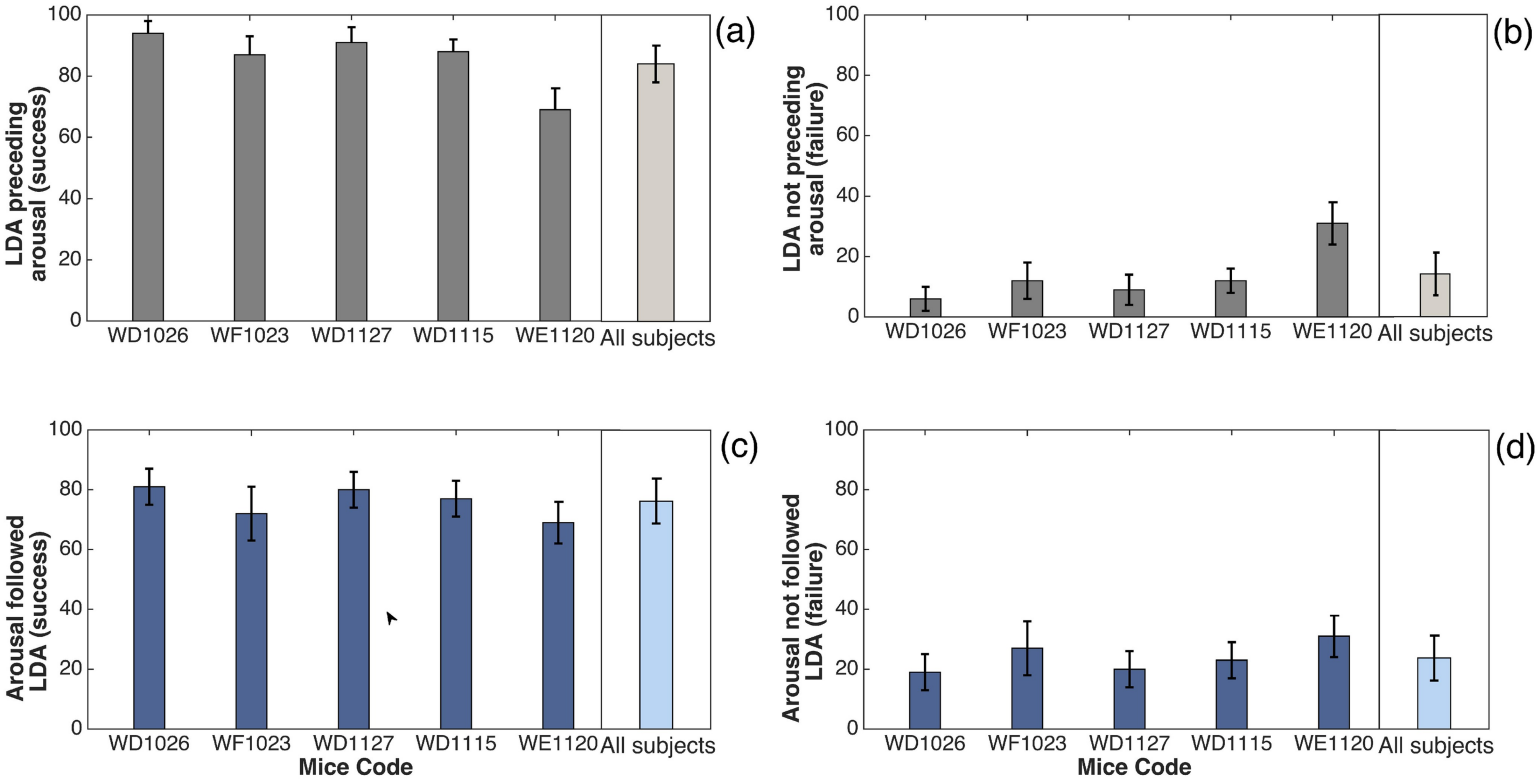
Statistical results of LDA events and arousals from accelerometer records (AR). In panels (a) and (b) we observe that LDA is related to a following arousal; (c) and (d) display arousal events related to a previous LDA occurrence. Here we exhibit data separately for each animal (all data here is related to Table 1), and also the average of all subjects shown in the last column of each panel. We point the LDA preceding arousal events (a) as successes (true positives), and (b) as a failures (false positives) for each animal. Moreover, the cases in which arousals are preceded by LDA are disclosed in (c) as successes, and (d) as failures. The error bars in the figure were calculated using the standard error $\sqrt{\frac{p(1-p)}{n}}$ for *p* the respective probability and *n* the sampling number.

These descriptive statistics are summarized in Table 1. In addition, the graphic representation (in bars) of AR fluctuation related to the temporal DET analysis described in Table 1 is represented in Fig 4. The success estimation, with an LDA preceding an arousal, ranges from 69% to 94%, according to the animal, while an arousal followed by LDA ranges from 68% to 81%.

**Table 1:** Summary of the descriptive statistic of experimental results of LDA events and arousals from Accelerometer records (AR).

Statistic Events Name	Mice Code				
	WD1026	WF1023	WD1127	WD1115	WE1120
LDA preceding by Arousal (success)	94% ± 4%	88% ± 6%	91% ± 5%	88% ± 4%	71% ± 7%
LDA not preceding by Arousal (failure)	6% ± 4%	12% ± 6%	9% ± 5%	12% ± 4%	29% ± 7%
Arousal followed by LDA (success)	81% ± 6%	72% ± 9%	80% ± 6%	77% ± 6%	69% ± 7%
Arousal not succeeded by LDA (failure)	19% ± 6%	27% ± 9%	20% ± 6%	23% ± 6%	31% ± 7%
Precursor Time (s)	4.0 ± 2.8	4.7 ± 4.0	3.2 ± 2.3	4.9 ± 4.0	5.1 ± 4.8
Time between successive LDA (s)	79 ± 22	69 ± 26	63 ± 27	69 ± 25	74 ± 45
Time between successive arousal (s)	64 ± 16	58 ± 17	69 ± 37	65 ± 29	86 ± 42

The time unit is defined in seconds (s). All results are presented in the form: average and standard deviation. For a graphical view of the statistical information in this table see Fig 4.

Table 1 also indicates the average durations involved in the analysis: the precursor time, the time between two consecutive arousals and the time between two consecutive LDA events. The average precursor time, as well as its standard deviation have the same order of magnitude around 4 seconds. The average time between arousals is (73 ± 29)*s*, while the average between two consecutive LDA is (66 ± 30)*s*, we notice that both have the same order of magnitude around 1 min. These data match with the result is found in [31].

**Fig 5:**
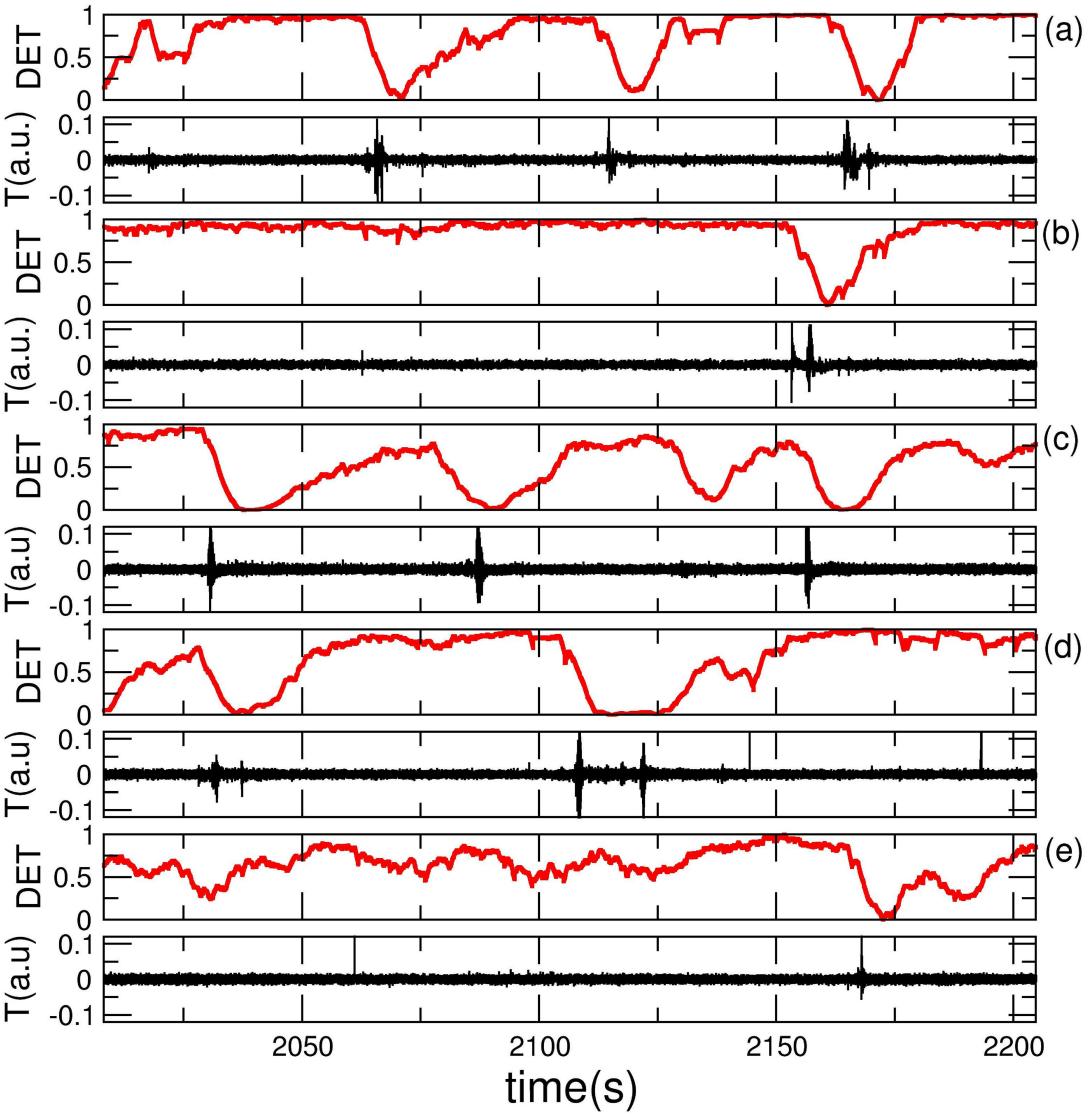
Typical AR signal with respective DET analysis for distinct animals. In all cases the signal are extracted randomly from SWS episodes. The arousals in panels (a), (b), (d) and (e) are successfully predicted, it means the LDA preceding arousal (success). In panel (c) the third LDA event is not followed by an arousal (failure). The two peaks in the second arousal from (d) are interpreted as a train of peaks in a single arousal event. The time scale in the legend was omitted despite all AR intervals have the same duration. Statistics involving all events about AR activity are shown in Table 1. Some combined statistical analysis using Venn Diagram are shown in Fig 6.

**Fig 6:**
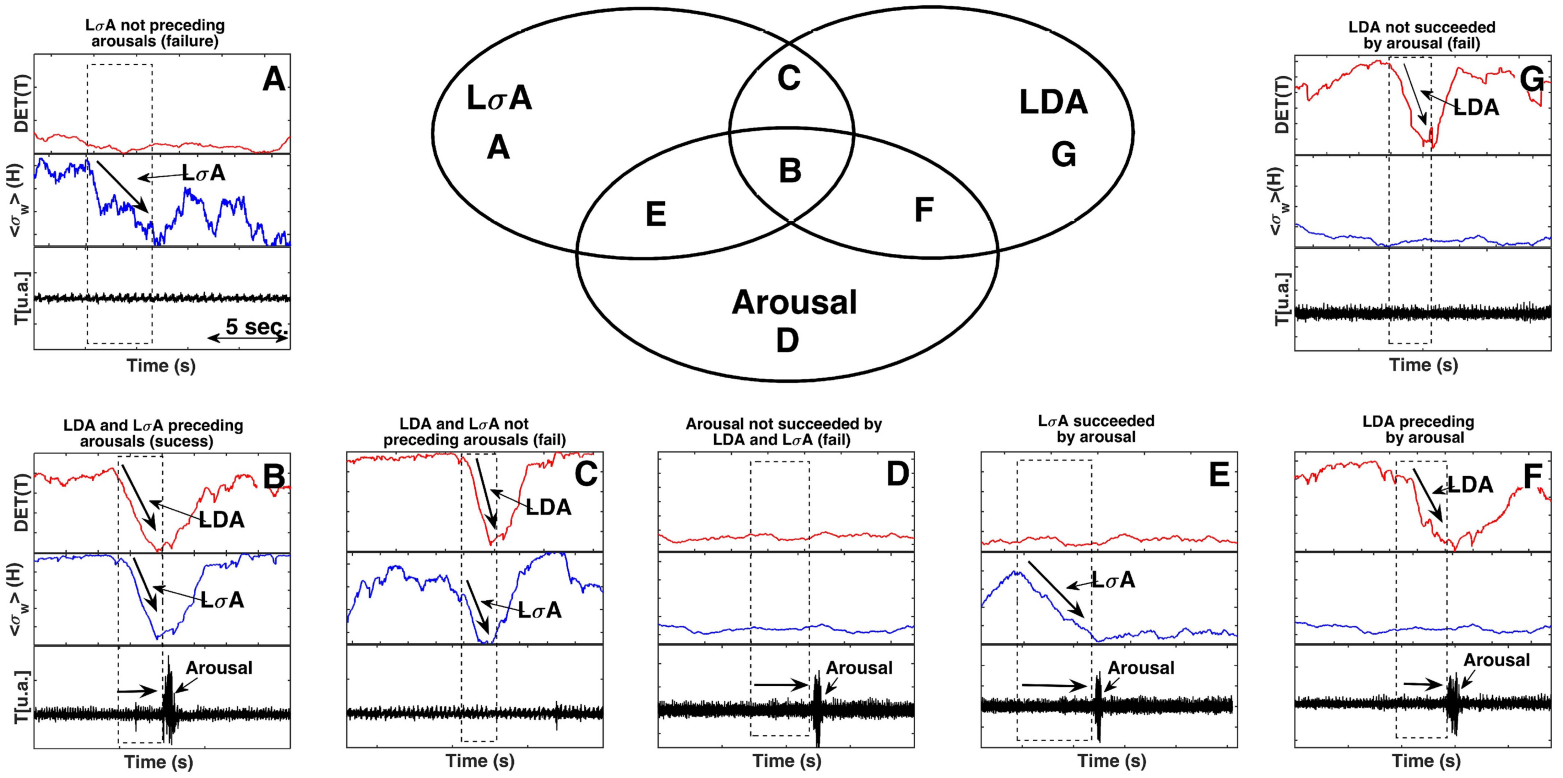
Venn Diagram representing events as arousal, LDA and L*σ*A phenomena. The figure shows the Venn diagram over all statistics that correlates the three phenomena: (i) large decreasing in determinism of accelerometer (LDA) in red; (ii) large decreasing in the fluctuation amplitude of LFP—CA1 (L*σ*A) in blue; and (iii) arousal episodes in black. The case where only one of those cited events—L*σ*A, arousal or LDA—appears is shown respectively in (A), (D) and (G) panels. We emphasize that these occurrences are rare. The most important case is (B) in which all three events take place simultaneously (true positive for both cases LDA and L*σ*A). The situation with L*σ*A preceding arousal is shown in panel (E), while LDA preceding arousal is in (G). Finally, the case with both LDA and L*σ*A without arousal is presented by panel (C) (false positive for both cases LDA and L*σ*A). The Venn diagram shows all possible events combined.

In Fig 5 we display AR and DET for five randomly selected episodes during SWS. We choose one sample for each analyzed individuals. For convenience all AR have the same duration. In agreement with the Venn Diagram presented in Fig 6, we observed that most of arousals displayed in this set of figures are preceded by abrupt decrease in DET (LDA-success or true positive). An exception of success disclosed here (an example of false positive—failure) is the third LDA event (from a total of four events) in Fig 5(c). It is worth of note that two peaks in the second arousal of panel (d) are interpreted as a single arousal event; indeed in some cases, changes in AR are not identified by a single peak in the AR, but appear as a train of peaks, which are considered as belonging to the same arousal event.

**Fig 7:**
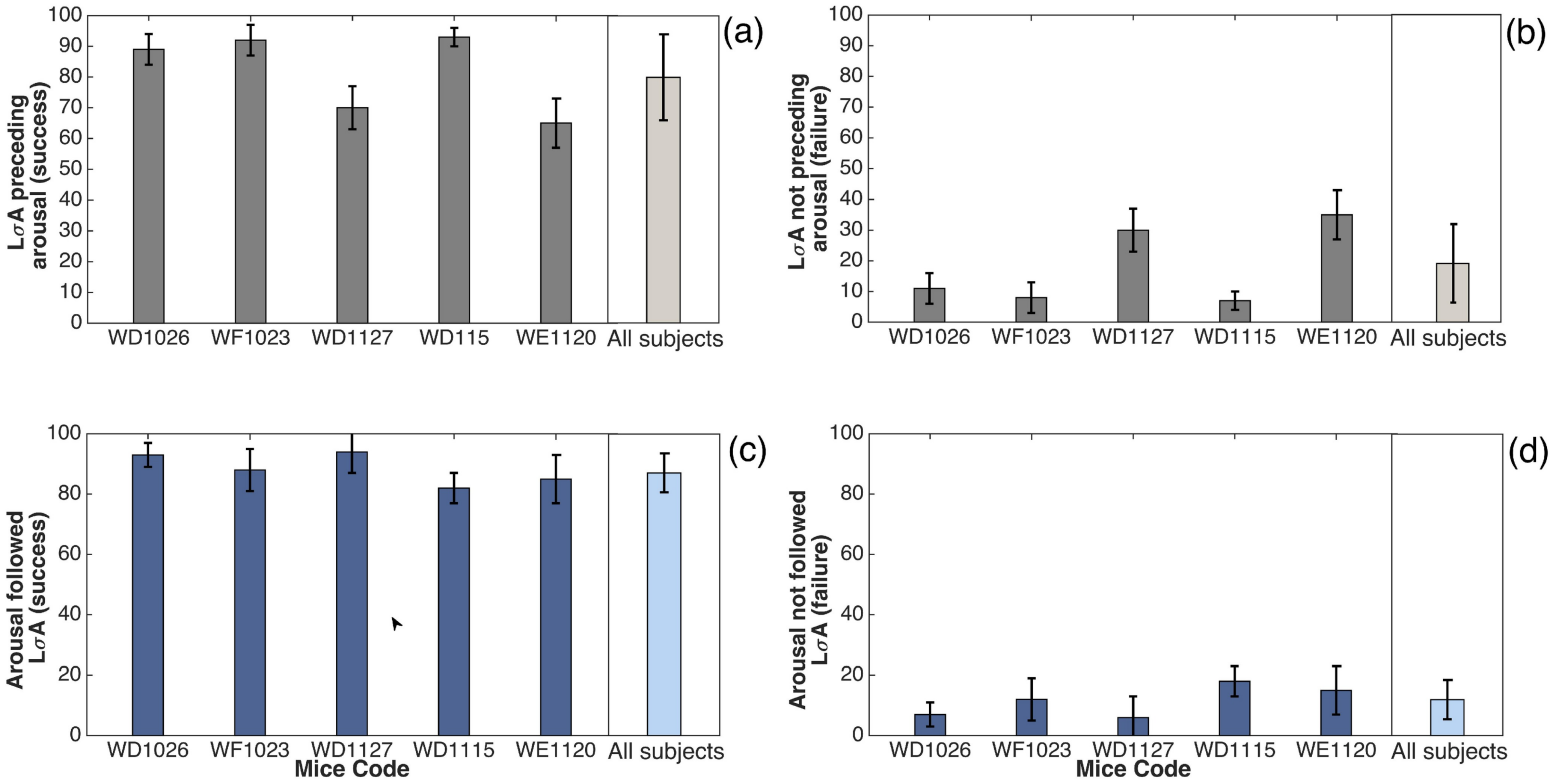
Statistical results of L*σ*A and arousal events from Hippocampus LPF CA1 region records related to the windowed variance analysis. In figures (a) and (b) we observe L*σ*A related to a following arousal; and in (c) and (d) panels display the arousal event related to a previous L*σ*A occurrence. Here we exhibit data separately for each animal (all data here related to Table 2), and else the average of all subjects shown in the last column of each panel. We indicate that the L*σ*A preceding arousal events in (a) as successes (true positive), and in (b) as failures (false positive). Moreover, the cases that arousals are preceding by L*σ*A are shown in (c) as success, and (d) as failure. The error bars in the figures were calculated using the standard error $\sqrt{\frac{p(1-p)}{n}}$ for *p* the respective probability and *n* the sampling number.

We perform similar statistics starting from hippocampal LFP activity to compare with AR detected arousal dynamics. The LFP amplitude decreasing was used here as a gold standard indication of the arousal, that means, arousals are associated with desynchronization of brain waves captured by a change in L*σ*A. In the same way as before, we associate L*σ*A with arousals. Those statistical results are shown in Table 2. The success over all individuals of hippocampus LFP activity in predicting arousal range from 65% to 92%. In addition, the case of arousal preceded by a decreasing in hippocampus LFP activity ranges from 82% to 94%. The statistics of the two tables (independently of each other) suggests that the predictability of arousal measured by *σ*_*W*_ of LFP signal and DET of AR are similar and almost synchronized in time. In addition, a graphic illustration of correspondence between arousals and windowed variance analysis of LFP activity, are illustrated in Fig 7. These associations plotted as bars are related to the statistics presented in Table 2.

**Table 2:** Summary of the statistical experimental results of L*σ*A and arousal events from Hippocampus LPF CA1 region records.

Hippocampal Statistic Events	Mice Code				
	WD1026	WF1023	WD1127	WD1115	WE1120
L*σ*A preceding Arousal (success)	89% ± 5%	92% ± 5%	70% ± 7%	93% ± 3%	65% ± 8%
L*σ*A not preceding Arousal (failure)	11% ± 5%	8% ± 5%	30% ± 7%	7% ± 3%	35% ± 8%
Arousal followed by L*σ*A (success)	93% ± 4%	88% ± 7%	94% ± 7%	82% ± 5%	85% ± 8%
Arousal not succeeded by L*σ*A (failure)	7% ± 4%	12% ± 7%	6% ± 7%	18% ± 5%	15% ± 8%

All results are presented in the form of percentage as followed: average and standard deviation. For a graphical view of the statistical information in this table see Fig 7.

From these results, we point out that the advantage of using the DET of the AR in analyzing the sleep behavior over intra-cranial LFP signal are: the technique is more convenient, simpler and the process is non-invasive. We shall discuss these points in the following section.

## Discussion

This work explores subtle changes in mice body activity during SWS sleep state. The sleep time between arousal episodes reveals a typical duration around 1 min. Past works focus on descriptive statistics of arousal duration as well as the time interval between them [6]. Some studies have shown that duration of arousal episodes [9] follow a power-law with the same slope for mice, rats, cats and humans [31]. However, the duration of one sleep episode between two consecutive arousals follows exponential-like distribution with a characteristic time-scale. In fact, the characteristic time of this exponential curve, that defines a typical sleep duration, depends on the species [31] and of the sleep stage.

The recurrence analysis of AR data determinism during SWS usually displays an unique dynamical behavioral pattern. The typical DET behavior is characterized by three distinct stages: first, a progressive increase, followed by a stationary plateau and finally an abrupt decrease that occurs immediately before the arousal event. In other words, in SWS stage, during two consecutive arousal episodes, the DET increases until it reaches a critical threshold and then quickly goes to a vanishing zero. The sharp decrease is associated with the arousal, a mechanism that can be used as an arousal prediction tool.

Our analysis of AR corroborates with a cyclic alternating process (CAP) hypothesis in sleep, as proposed in the eighties [32]. In the original work [32], performed in humans, CAP is a long-lasting periodic activity consisting of two alternate EEG patterns related to arousal periodicity. In this study, the CAP process is characterized by a specific sequence of events including a K-complex pattern and episodes of alpha waves observed in the EEG signal. Our work, performed in mice, identify similar cycles related to arousal onset in hippocampal LFP fluctuations, and, additionally, in the accelerometer data.

This work describes, for the first time, an evidence that body movements manifest a precursor behavior preceding arousal episodes. The increase of DET of AR signal, during SWS stage, and its abrupt decrease few second before the arousal, suggest that the temporal signal of AR probably is more rhythmic and slower during SWS than non-SWS states. As previously shown [17], AR can express breathing movement, heart beating, tremors or limb movement. Following previous results about amplitude of AR data [17], we argue that the these physiological events could be specially related to respiratory behavior. We believe that the more regular and deep respiratory activity during the SWS sleep stage is associated with a the signal determinism.

Furthermore, following a typical negative feedback, the dynamics of blood gas concentration, specially the rise in carbonic acid, induces some neural activation signals that stimulate the neural respiratory center in the brain-stem, such as Boltzinger and pre-boltzinger nuclei [33], which, in the studied cases, could produce remarkable changes in respiratory rhythm that cause abrupt decrease in the DET. Simultaneously, activation signal from brain-stem could also induce electrical desynchronization in high neural centers, such as the hippocampus and/or cortex brain areas. Both phenomena could together trigger a command for arousal initiation on the primary motor cortex.

In this way, we hypothesize that the micro-arousals detected by the AR in SWS stage are determined by a neural process primarily induced by respiratory centers localized in brain-stem. Two studies [34–36] have shown that it is possible to detect accurately changing in breathing condition from temporal-spatial dynamical of EEG signal in humans. Another study has proposed the use of accelerometer plus oximeter implanted in smart-phone device to detect obstructive sleep apnea [37]. Additionally, respiratory signal can be derived from EEG and it has been suggested the use of this method as a promising application in medical field [38].

The difference of our system and the references cited in the previous paragraph is the high sample rate in AR and the DET as measure of arousal predictability. Taking these studies into account, we suggest that AR data could be explored in humans to investigate a probable association with arousal detection as found here in mice. Accelerometer data, linked to temporal series analyses, could provide an important toll for many sleep and movement disorders, related to either its diagnosis or prevention. More specifically, the use of determinism in accelerometer analysis could also be very helpful in the search for an early diagnose tool in progressive neuro-degenerative disorders, such as Parkinson disease and in Parkinson rodent models: in these situations, subtle movement alterations, not detected by human eye or common senses, could be detected through alterations in determinism fluctuation. For the last, in animal analysis, this approach could be used to distinguish different sleep phases, which may include the separation between superficial and deep slow-wave sleep in rodents.

## Conclusion

The fact that a maximum in the determinism of AR signal is attained before arousal events suggests that we can use this methodology as a convenient and non-invasive precursor of arousal in SWS phase. In this way, the use of determinism as a mathematical tool of analysis for arousal predictability represents a promising technique for sleep apnea diagnostic and treatment as well as for neuro-degenerate diseases with sleep disturbances. Those subtle movement patterns given by AR data, which occurs at SWS stage, are commonly not detectable by eye perception. We believe that, pairing AR signal with EEG recordings and careful behavior observation can aggregate remarkable information about many of the still unknown mechanisms associated with generation of subtle arousal episodes in sleep.

## Supporting information

S1 Dataset Raw data archive from an entire hour of mouse WD1026. The four columns represent, respectively, raw data from accelerometer, LFP from hippocampus, LFP from sensorial primary cortical area S1 and LFP from motor primary cortex area M1. Here we took the third registered hour from light phase. Data was acquired at a 1000Hz sample rate.
(ZIP)

S2 Dataset Raw data archive from an entire hour of mouse WF1023. The four columns represent, respectively, raw data from accelerometer, LFP from hippocampus, LFP from sensorial primary cortical area S1 and LFP from motor primary cortex area M1. Here we took the third registered hour from light phase. Data was acquired at a 1000Hz sample rate.
(ZIP)

S3 Dataset Raw data archive from an entire hour of mouse WD1127. The four columns represent, respectively, raw data from accelerometer, LFP from hippocampus, LFP from sensorial primary cortical area S1 and LFP from motor primary cortex area M1. Here we took the third registered hour from light phase. Data was acquired at a 1000Hz sample rate.
(ZIP)

S4 Dataset Raw data archive from an entire hour of mouse WD1115. The four columns represent, respectively, raw data from accelerometer, LFP from hippocampus, LFP from sensorial primary cortical area S1 and LFP from motor primary cortex area M1. Here we took the third registered hour from light phase. Data was acquired at a 1000Hz sample rate.
(ZIP)

S5 Dataset Raw data archive from an entire hour of mouse WE1120. The four columns represent, respectively, raw data from accelerometer, LFP from hippocampus, LFP from sensorial primary cortical area S1 and LFP from motor primary cortex area M1. Here we took the third registered hour from light phase. Data was acquired at a 1000Hz sample rate.
(ZIP)
